# Obituary

**Published:** 1872-11

**Authors:** 


OBITUARY.—We are pained to learn of the death of Professor James Hadley, of Yale College, brother of our distinguished townsman, Professor George Hadley. To show the appreciation in which Prof. Hadley was held, we quote the following from the *N. Y. Evening Post:* "Prof. James Hadley was one of the best scholars America has ever produced, although comparatively still a young man. He was a scholar above and beyond all things else, and, what is strange in this country, where it is customary to bring every mental attainment before the public in search of a market of some kind, he seemed never to have appreciated the power of public opinion in respect to reputation, but studied simply for study's sake. His public work was all confined to New England, so far as we remember, and consisted of a number of papers, mainly on philological topics, in the *New Englander*, and an appreciative criticism grounded on a careful comparison of the translation with the text, of Mr. Bryant's version of " Homer's Iliad, in the *College Courant*. Aside from this he used his store of knowledge solely for the good of his college, any chair in which, it was said of him, he was competent to fill. Dr. Woolsey believed him to be one of the best, if not the best scholar in the country."

Dr. O. K. Parker, of Clarence, Erie county, N. Y., died at his residence, Nov. 16th, at the age of 47. Dr. Parker's death will be a loss to the profession of Erie county which cannot be easily replaced. We shall in our next issue publish resolutions which were adopted at a special meeting of the Erie County Medical Society on the death of Dr. Parker.

